# Association of plant-based diets with adropin, atherogenic index of plasma, and metabolic syndrome and its components: A cross-sectional study on adults

**DOI:** 10.3389/fnut.2023.1077709

**Published:** 2023-04-11

**Authors:** Farnaz Shahdadian, Parvane Saneei, Keyhan Lotfi, Awat Feizi, Gholamreza Askari, Sayyed Morteza Safavi

**Affiliations:** ^1^Department of Clinical Nutrition, Nutrition and Food Security Research Center, School of Nutrition and Food Science, Isfahan University of Medical Sciences, Isfahan, Iran; ^2^Department of Community Nutrition, Nutrition and Food Security Research Center, School of Nutrition and Food Science, Isfahan University of Medical Sciences, Isfahan, Iran; ^3^Department of Community Nutrition, School of Nutritional Sciences and Dietetics, Tehran University of Medical Sciences, Tehran, Iran; ^4^Department of Biostatistics and Epidemiology, School of Public Health, Isfahan University of Medical Sciences, Isfahan, Iran

**Keywords:** plant-based diet indices, metabolic syndrome, atherogenic index of plasma, adropin, cross-sectional study

## Abstract

**Background:**

Little is known about the association of plant-based diet indices with metabolic syndrome (MetS) and its novel predictive biomarkers, including the atherogenic index of plasma (AIP) and adropin. We aimed to investigate the association of plant-based diets with adropin, atherogenic index of plasma, and MetS and its components in adults.

**Methods:**

The present population-based cross-sectional study was conducted on a representative sample of adults aged 20–60 years in Isfahan, Iran. Dietary intake was obtained through a validated 168-item semi-quantitative food frequency questionnaire (FFQ). Peripheral blood was obtained after an overnight fast of at least 12 h from each participant. MetS was identified based on the Joint Interim Statement (JIS). AIP was calculated as a logarithmically transformed ratio of triglyceride (TG)/high-density lipoprotein cholesterol (HDL-c), and serum levels of adropin were measured by an ELISA kit.

**Results:**

A total of 28.7% of subjects had MetS. No significant association was found between the overall plant-based diet index (PDI) and the healthful plant-based diet index (hPDI) with MetS. However, a non-linear association was observed between hPDI and MetS. Subjects in the third quartile of the unhealthful plant-based diet index (uPDI) had higher odds of MetS compared to the first quartile (OR: 2.39; 95% CI: 1.01, 5.66). The highest quartile of PDI (OR: 0.46; 95% CI: 0.21, 0.97) and the third quartile of hPDI (OR: 0.40; 95% CI: 0.18, 0.89) were associated with decreased odds of having high-risk AIP compared to the first quartile, after adjusting for potential confounders. No linear association was found between quartiles of plant-based diet indices and serum levels of adropin.

**Conclusion:**

Plant-based diet index (PDI) and hPDI were not associated with the prevalence of MetS in adults, while moderate adherence to uPDI increased the prevalence of MetS. In addition, high adherence to PDI and moderate adherence to hPDI were associated with decreased odds of high-risk AIP. No significant association was found between plant-based diet indices and serum adropin levels. To confirm these findings, further studies with prospective designs are warranted.

## Introduction

Metabolic syndrome (MetS) is a condition defined by a cluster of metabolic disorders, including impaired fasting glucose, high blood pressure, abdominal obesity, and dyslipidemia [hypertriglyceridemia and low high-density lipoprotein cholesterol (HDL-c) levels] (1, 2). The worldwide prevalence of MetS in the adult population ranges from 20 to 25% (3), with a growing trend in both developing and developed countries (4–6). This public health problem has been considered an important risk factor for developing type 2 diabetes mellitus (T2DM), cardiovascular diseases (CVDs), as well as all-cause mortality (7, 8). Although the exact underlying pathophysiology of MetS has not been clearly determined, abdominal obesity and insulin resistance as the results of a sedentary lifestyle and unhealthy eating patterns might play key roles in developing MetS (9, 10).

Recently, some biomarkers including adiponectin, leptin, resistin, apelin, and irisin might serve as MetS predictors (11, 12). One of the novel biomarkers that might contribute to MetS development is adropin. Adropin that contributes to nutrients and energy hemostasis is encoded by the energy homeostasis associated (Enho) gene and is expressed in the brain and the liver (13). Low serum adropin levels are associated with an increased risk of T2DM, endothelial dysfunction, obesity, and MetS (14–16). Another novel predictive biomarker of obesity and MetS is the atherogenic index of plasma (AIP) (17, 18). The sensitivity of AIP to reflect the interaction of protective lipoproteins with atherogenic ones is higher than the other atherogenic indices (17). Previous studies reported that elevated levels of AIP are associated with higher waist circumference and increased risk of chronic diseases (19, 20). In addition, AIP has been considered a strong predictor for developing MetS (17).

Environmental factors, including eating patterns, might be associated with the prevalence of MetS and related indices. Ganesh Kumar et al. reported that a low-carbohydrate high-fat diet compared to a high-carbohydrate low-fat diet increased the serum adropin levels in mice; they additionally reported that diet-induced obesity and overnight fasting conditions might suppress the serum levels of adropin (21). Recent studies reported that healthy lifestyle behaviors, including healthy eating patterns and physical activity, were associated with low levels of AIP (22, 23). In the case of MetS, previous studies suggested that adherence to the Dietary Approaches to Stop Hypertension (DASH) and Mediterranean diets decreased, and the animal-based diets increased the risk of MetS (24–26). In addition, previous studies reported that healthy plant foods including whole grains, vegetables, fruits, and nuts were associated with a lower risk of MetS. However, some less healthy plant foods such as refined grains and sugar-rich plant foods were associated with a higher risk of MetS (27, 28). This difference between plant foods and their association with the risk of disease is reflected in a graded scoring system named plant-based diet indices.

Plant-based diet indices include three indices as follows: an overall plant-based diet index (PDI) which represents the intake of all plant food with decreasing the consumption of animal food. A healthful plant-based diet index (hPDI) represents the consumption of healthy plant foods; and an unhealthful plant-based diet index (uPDI) represents the intake of less healthy plant foods (29). Previous studies demonstrated that hPDI was associated with a lower risk of chronic diseases, while adherence to uPDI was associated with a greater risk of chronic diseases (27–30).

No previous study has evaluated the association of plant-based diet indices with adropin and AIP, and a limited number of studies examined the association between plant-based diet indices and metabolic syndrome, especially in Middle Eastern countries. Therefore, we aimed to investigate the association of plant-based diets with adropin, atherogenic index of plasma, and metabolic syndrome and its components in adults.

## Materials and methods

### Study design and population

The present population-based cross-sectional study was conducted on a somehow representative sample of adults aged 20–60 years in Isfahan, Iran, in 2021. Isfahan was considered as big city located in the center of Iran, with six educational districts consisting of 285 primary and secondary schools. Three or four schools were chosen across each district, and 20 schools were totally defined as our final sample location, using a stratified multistage random cluster sampling method. After that, information on the study research was sent to selected schools. After the agreement of the administrators of the schools, the subjects that consented to take part in the study were recruited. To attain a relatively representative sample of the general population, all adults who were working in the selected schools, such as employees, teachers, school managers, assistants, and crews, were included in the present studies. Subjects with a history of diseases, including CVD, stroke, type 1 diabetes, and cancer, or those who used a special diet during the last 6 months, were pregnant or lactating were not included in the current study.

Since no previous study has evaluated the association between adropin and dietary indices, we used irisin (as a similar analogous factor for adropin) to calculate the sample size of the study (31, 32). Considering a power of 80%, alpha error of 0.1, and a correlation coefficient of 0.1 for the association of irisin with dietary indices, the sample size was calculated to be at least 450 subjects; by taking a low response rate into account, a total of 600 subjects were invited to the current study. Finally, a total of 527 adults were included in the current study for metabolic syndrome and AIP analysis, and 497 subjects were included for adropin-related analysis. The Ethics Committee of the Isfahan University of Medical Sciences approved the protocol of the study (no: IR.MUI.RESEARCH.REC.1400.370), and all participants signed a written informed consent.

### Dietary intake assessment

A validated 168-item semi-quantitative food frequency questionnaire (FFQ) was used to evaluate usual dietary intake during the last year in the study population (33). Based on the standard protocol, a trained dietitian instructed the participants on how to complete this self-administered dietary questionnaire. Participants were asked how often they consumed food items on the basis of 10 categories of frequency (“seldom/never,” once per month, 2–3 per month, once per week, 2–3 per week, 4–6 per week, 1 per day, 2–3 per day, 4–5 per day, and 6 or more per day). In addition, they were asked to report the portion sizes of each food and beverage item. By using household measurements, the frequency of consumption was changed to daily intake, and portion sizes were converted to grams. Finally, the food intake (g/day) was transformed to Nutritionist IV software (version 7; N-squared computing, OR, United States), to compute the total energy and nutrient intake.

### Plant-based diet indices

Using the dietary intake data, three types of plant-based diet indices including PDI, hPDI, and uPDI were created through the use of the method proposed by Satija et al. (29). In brief, all foods were divided into 18 groups according to similarities of nutritional and culinary characteristics. These 18 food groups belonged to broader categories of healthy plant food groups (whole grains, fruits, vegetables, nuts, legumes, vegetable oils, and tea and coffee), less healthy plant food groups (fruit juices, refined grains, potatoes, sugar-sweetened beverages, sweets, and desserts), and animal food groups (animal fats, dairy, eggs, fish, meat, and miscellaneous animal-based foods; Supplementary Table 1). On account of changing the fatty acid profile over time for margarine and hydrogenated vegetable oils, these two items were not included in the indices calculation, and instead, we made an adjustment for them in multivariable models. Because of the lack of accurate reporting due to limitations in alcohol consumption in the Iranian population, this item was not considered in the current analysis. To calculate three indices, the food groups were first adjusted for energy intake using residual methods (34, 35). A total of 18 energy-adjusted food groups were ranked into quintiles, and positive or reverse scores were assigned to them. For positive scores, subjects in the lowest quintile of food group consumption were given a score of 1, whereas those in the highest quintile were given a score of 5. For reverse scores, subjects in the lowest quintile of food group consumption received a score of 5, whereas those in the highest one received a score of 1. For the PDI, both healthy and unhealthy plant foods received positive scores. However, for the hPDI and uPDI, only healthy plant foods and unhealthy plant foods were given positive scores, respectively. In all three indices, animal food groups were assigned reverse scores (Supplementary Table 1). All plant-based diet indices theoretically ranged from 18 to 90 and higher scores were associated with greater adherence to the diet index (29).

### Anthropometric and blood pressure measurement

Weight measurement was conducted using the body composition analyzer (Tanita MC-780MA, Tokyo, Japan) with 0.01 kg accuracy, while participants were minimally clothed without shoes. Height was measured using a non-stretch tape to the nearest 0.1 cm while subjects unshod. Body mass index (BMI) was computed as weight (kg)/height^2^ (m^2^). In addition, waist circumference (WC) was assessed to the nearest 0.1 cm at the midway level between the lower rib margin and the iliac crest at the end of exhalation in standing positions and without any pressure on the body surface.

Arterial blood pressure (BP) was measured two times, while subjects were seated comfortably after a resting period of at least 5 min. The participants were asked to be overnight fast and refrain from smoking and exercise for at least half an hour before BP measurement; if the bladder was full, it should be emptied. Systolic and diastolic BPs (SBP and DBP) were measured by a digital sphygmomanometer (OMRON, M3, HEM-7154-E, Japan), with an accuracy of 0.5 mmHg, on the left arm. BP was defined as the mean of the first and second measurements (36).

### Assessment of biochemical parameters

A measure of 10 ml of peripheral blood was obtained using venipuncture, after an overnight fast of at least 12 h from each participant. The serum was separated by centrifugation at 3,500 rpm for 10 min. Biochemical parameters [including fasting blood glucose (FBG), serum levels of total cholesterol, HDL-c, low-density lipoprotein cholesterol (LDL-c), and serum triglyceride (TG)] were measured by the enzymatic colorimetric method using BioSystem Kit Company on Biosystem A15 autoanalyzer. AIP was calculated as a logarithmic transformation of the ratio of TG to HDL-c (37). ZellBio GmbH ELISA Kit (Germany) was used for the quantitative assay of human adropin on the basis of the Biotin double antibody sandwich technology. The assay range of adropin ranged from 30 to 960 pg/mL with 4 pg/mL sensitivity; the intra-assay and inter-assay CV were <10 and <12%, respectively.

### Definition of MetS

The Joint interim statement (JIS) was applied to define MetS. Subjects who met at least three of the following conditions were considered as MetS: (1) elevated WC (WC ≥ 94 cm in men and ≥80 cm in women), (2) elevated TG [TG ≥ 150 mg/dl (1.7 mmol/L) or on drug treatment for elevated triglycerides], (3) hypertension (SBP ≥ 130 mmHg or DBP ≥ 85 mmHg or on antihypertensive drug treatment in a patient with a history of hypertension), (4) hyperglycemia (FBG ≥ 100 mg/dl or on drug treatment for elevated glucose), and (5) reduced HDL-c [HDL-c <40 mg/dl (1.03 mmol/L) in men and <50 mg/dl (1.3 mmol/L) in women] (38).

### Covariates assessment

Demographic and socioeconomic characteristics of the study population were evaluated by self-reported questionnaires. In addition, to evaluate the physical activity, the International Physical Activity Questionnaire Short Form (IPAQ-SF) was applied, and subjects were divided into three categories including inactive, minimally active, and health-enhancing physical activity (HEPA) (39).

### Statistical analysis

To evaluate the normality of quantitative variables, the Kolmogorov–Smirnov test was applied. The quantitative variables were illustrated as mean ± SD/SE and qualitative variables as frequency (percentage). To compare the quantitative variables across quartiles of plant-based diet indices, a one-way analysis of variance (ANOVA) was applied, while for categorical variables, the chi-square test was used. In addition, the independent sample *t*-test was applied to examine the quantitative variables between subjects with and without metabolic syndrome. The general linear model was applied to evaluate the age, sex, and energy-adjusted means of nutrient intake by quartiles of the plant-based diet indices. To determine the association of plant-based diets with MetS, its component, and high-risk AIP (values greater than the median), multivariable logistic regression was applied. The odds ratios (ORs) and their 95% confidence intervals (95% CIs) were calculated in crude and adjusted models. Potential confounders were selected based on the previous literature (27, 40). In Model I, adjustment was made for the main confounders (age, sex, and energy intake). In Model II, additional adjustments were conducted for education status, smoking status, marital status, socioeconomic status (SES), physical activity, and intake of margarine and hydrogenated vegetable oils. In Model III, BMI was added to previous adjustments. The first quartile of plant-based diet indices was considered the reference category in all analyses. In addition, a crude and multivariable-adjusted linear regression model was applied to predict serum adropin levels. To evaluate the non-linear association, a restricted cubic spline regression analysis was conducted. SPSS software version 20 (IBM, Chicago, IL) and STATA version 14 were used to perform analysis, and a *P*-value of <0.05 (two-tailed) was considered statistically significant.

## Results

### Study population characteristics

**Figure 1:**
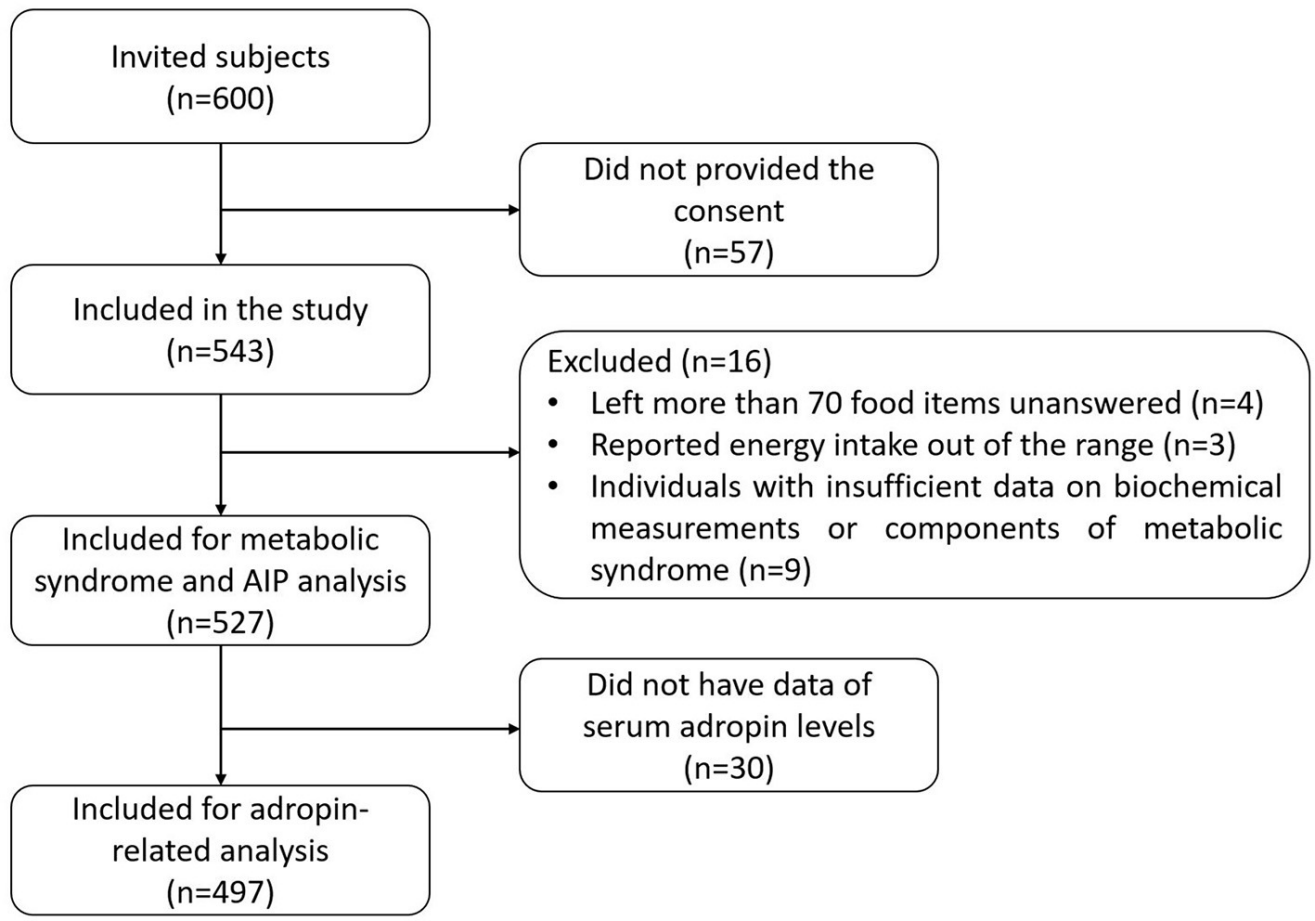
The study participant flow chart.

In total, 600 subjects were invited to the current study. Among 600 invited individuals, 543 subjects provided their consent. Subjects who had left more than 70 food items unanswered (*n* = 4) reported energy intake out of the range of 800–4,200 kcal (41) (*n* = 3), and individuals with insufficient data on biochemical measurements (*n* = 1) or components of metabolic syndrome (*n* = 8) were excluded from the analysis. Finally, a total of 527 adults were included in the current cross-sectional study for metabolic syndrome and AIP analysis. In the case of adropin, 30 subjects did not have data on serum adropin levels; therefore, 497 subjects were included for adropin-related analysis (Figure 1). The overall analysis was conducted on 527 adults with a mean age and BMI of 42.65 ± 11.18 years and 26.90 ± 4.43 kg/m^2^, respectively; 54.3% of included subjects were male. The prevalence of MetS and high-risk AIP were 28.7 and 49%, respectively. Demographic and cardiometabolic characteristics of the study participants, divided by quartiles of the plant-based dietary indices, are presented in Table 1. The mean age of subjects was higher in the upper quartile of PDI and hPDI compared to the first quartile, while it was lower in the top quartile of uPDI. The percentage of women in the fourth quartile of hPDI was more than the first quartile, while those in the fourth quartile of uPDI were more likely to be men. The percentage of married subjects in the highest quartile of uPDI was lower than the first one. Anthropometric measurements, education, SES, physical activity, MetS and its components, adropin levels, and AIP were not significantly different among the quartiles of the various types of plant-based dietary indices.

**Table 1:** Demographic and cardiometabolic characteristics of participants across energy-adjusted quartiles of the plant-based dietary indices^a^.

**Variables**	**PDI**		***P*-value^b^**	**hPDI**		***P*-value^b^**	**uPDI**		***P*-value^b^**
	**Quartile 1**	**Quartile 4**		**Quartile 1**	**Quartile 4**		**Quartile 1**	**Quartile 4**	
Sample size (*n*)	118	145		116	144		121	121	
Median score (range)	47 (34–49)	60 (58–71)		45 (28–48)	63 (59–81)		45 (35–48)	63 (60–76)	
Age (year)	40.48 ± 10.93	45.46 ± 11.14	<0.001	37.25 ± 10.29	47.18 ± 11.38	<0.001	44.14 ± 10.44	38.90 ± 11.18	<0.001
Body weight (kg)	77.92 ± 15.49	74.94 ± 15.18	0.12	75.95 ± 14.56	74.75 ± 15.42	0.80	74.66 ± 16.23	77.35 ± 15.20	0.51
BMI (kg/m^2^)	26.98 ± 4.44	26.82 ± 4.67	0.98	26.14 ± 4.22	27.00 ± 4.82	0.18	26.98 ± 5.15	26.67 ± 4.32	0.59
WC (cm)	93.69 ± 12.33	92.89 ± 11.18	0.58	91.70 ± 11.68	92.92 ± 11.86	0.79	91.57 ± 12.66	93.15 ± 12.07	0.69
Sex			0.56			0.04			<0.001
Male	68 (57.6)	77 (53.1)		71 (61.2)	67 (46.5)		49 (40.5)	83 (68.6)	
Female	50 (42.4)	68 (46.9)		45 (38.8)	77 (53.5)		72 (59.5)	38 (31.4)	
Education			0.34			0.27			0.24
Diploma or lower	11 (9.3)	14 (9.8)		13 (11.2)	12 (8.5)		8 (6.7)	18 (14.9)	
Higher than Diploma	107 (90.7)	129 (90.2)		103 (88.8)	130 (91.5)		111 (93.3)	103 (85.1)	
Marital status			0.83			0.03			0.01
Single	18 (15.3)	25 (17.4)		23 (20.2)	28 (19.6)		19 (15.8)	31 (25.8)	
Married	98 (83.1)	116 (80.6)		91 (79.8)	112 (78.3)		98 (81.7)	88 (73.3)	
Divorced or widow	2 (1.7)	3 (2.1)		0 (0.0)	3 (2.1)		3 (2.5)	1 (0.8)	
Smoking			0.49			0.24			0.32
Non-smoker	101 (94.4)	114 (92.7)		92 (92.0)	116 (92.8)		103 (93.6)	99 (93.4)	
Ex-smoker	2 (1.9)	5 (4.1)		2 (2.0)	5 (4.0)		5 (4.5)	1 (0.9)	
Smoker	4 (3.7)	4 (3.3)		6 (6.0)	4 (3.2)		2 (1.8)	6 (5.7)	
SES			0.43			0.32			0.06
Low	26 (32.5)	31 (33.3)		29 (35.4)	23 (27.7)		17 (20.7)	33 (41.8)	
Moderate	21 (26.3)	28 (30.1)		25 (30.5)	21 (25.3)		25 (30.5)	24 (30.4)	
High	33 (41.3)	34 (36.6)		28 (34.1)	39 (47.0)		40 (48.8)	22 (27.8)	
Physical activity			0.30			0.86			0.13
Inactive	61 (51.7)	91 (63.6)		65 (56.5)	80 (55.9)		59 (49.2)	65 (54.2)	
Minimally active	48 (40.7)	41 (28.7)		43 (37.4)	54 (37.8)		51 (42.5)	40 (33.3)	
HEPA active	9 (7.6)	11 (7.7)		7 (6.1)	9 (6.3)		10 (8.3)	15 (12.5)	
Metabolic syndrome	36 (30.5)	41 (28.3)	0.58	29 (25.0)	48 (33.3)	0.35	36 (29.8)	29 (24.0)	0.58
SBP (mmHg)	123.11 ± 16.53	121.52 ± 17.02	0.61	121.09 ± 15.91	123.41 ± 16.76	0.22	122.52 ± 16.29	120.67 ± 16.32	0.09
DBP (mmHg)	82.89 ± 9.62	83.19 ± 11.32	0.72	82.39 ± 9.10	83.12 ± 10.14	0.50	82.45 ± 8.93	82.22 ± 9.85	0.85
FBG (mg/dL)	91.75 ± 13.50	92.37 ± 18.96	0.96	90.48 ± 14.21	94.21 ± 23.74	0.41	95.08 ± 25.02	91.42 ± 18.07	0.33
TG (mg/dL)	154.32 ± 42.01	153.15 ± 42.69	0.93	154.88 ± 43.78	148.56 ± 36.44	0.37	148.99 ± 36.22	153.54 ± 38.99	0.54
TC (mg/dL)	187.01 ± 35.15	182.60 ± 30.42	0.60	180.75 ± 33.56	181.40 ± 32.99	0.30	182.28 ± 31.93	180.75 ± 30.40	0.46
LDL-C (mg/dL)	100.39 ± 31.52	95.60 ± 26.52	0.47	95.96 ± 28.60	95.91 ± 28.84	0.46	95.44 ± 28.12	95.49 ± 25.96	0.35
HDL-C (mg/dL)	55.75 ± 10.48	56.36 ± 10.16	0.38	54.27 ± 10.44	55.76 ± 10.01	0.27	57.04 ± 9.56	54.98 ± 9.46	0.21
Adropin (pg/ml)	54.35 ± 36.17	59.04 ± 43.36	0.41	57.68 ± 41.78	51.39 ± 25.13	0.08	55.51 ± 42.70	51.71 ± 24.15	0.11
AIP	0.43 ± 0.15	0.42 ± 0.15	0.71	0.44 ± 0.15	0.42 ± 0.12	0.31	0.41 ± 0.12	0.43 ± 0.13	0.25

a Quantitative variables: mean ± SD. Qualitative variables: frequency (percentage).

b Resulted from ANOVA for quantitative variables and chi-square test for categorical variables.

Multivariate adjusted dietary intakes across quartiles of the plant-based dietary indices are presented in Supplementary Table 2. The mean energy intake in the highest quartile of PDI was lower than the first one, while in hPDI and uPDI, the mean energy intake was higher in the highest quartiles. The mean intake of carbohydrates was greater, and the mean intake of proteins, fats, and cholesterol was lower in the highest quartile of the three types of plant-based diets compared to the first one. In addition, dietary fiber intake in the highest quartile of PDI and hPDI was greater than the first one. However, subjects in the highest quartile of uPDI had less intake of dietary fiber than those in the first quartile.

### Association of plant-based diets with MetS and high-risk AIP

**Figure 2:**
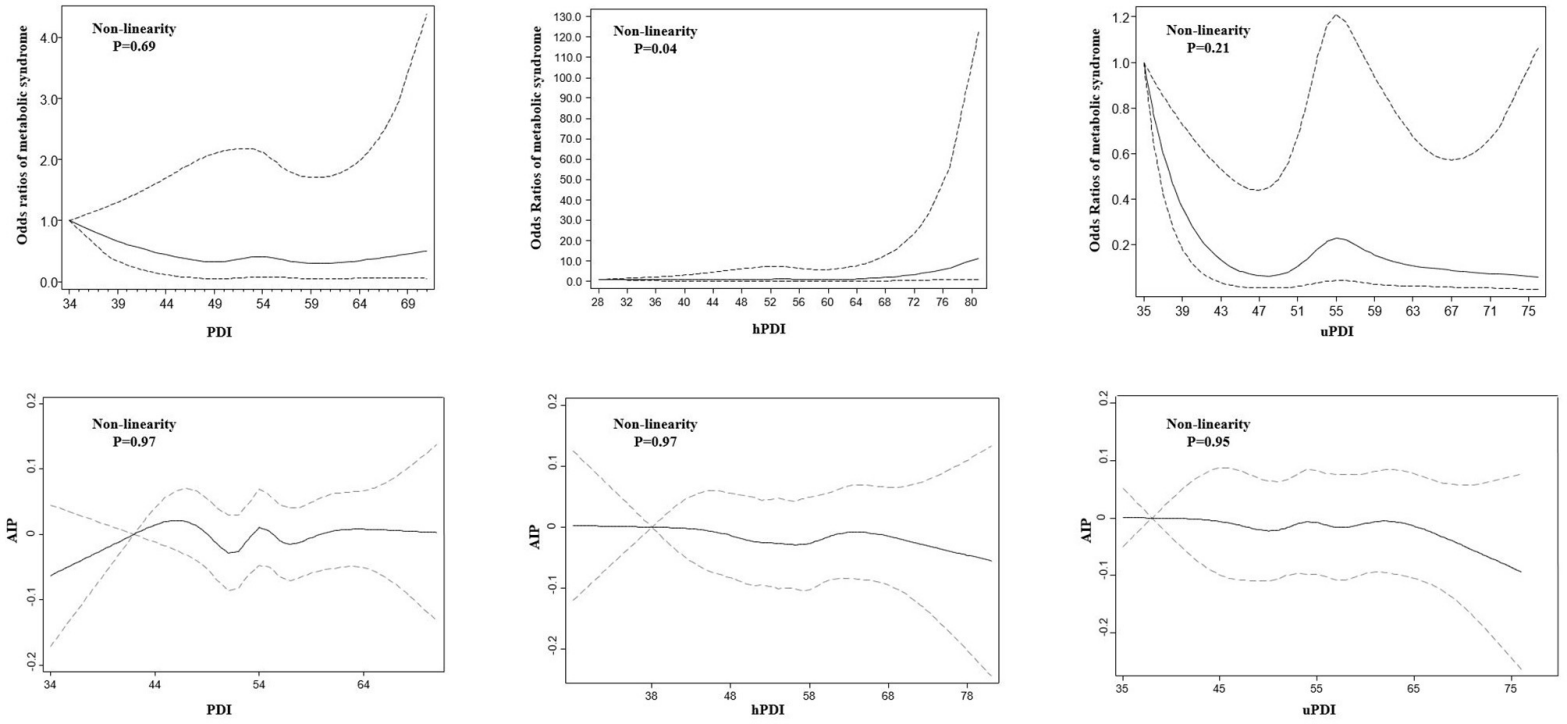
The non-linear association of all types of plant-based diet indices with MetS and AIP.

Multivariable-adjusted ORs (95% CI) for the association of plant-based diets with MetS and AIP are presented in Table 2. In the crude and adjusted models, no significant association was found between PDI and hPDI with MetS. Although no significant association was detected between uPDI and MetS in the crude model, after adjustment for potential confounders, the moderate adherence to uPDI (third quartile) was associated with an increased odds of MetS (OR: 2.39; 95% CI: 1.01, 5.66). In the case of the association of plant-based diets with high-risk AIP, the highest quartile of PDI was associated with decreased odds of high-risk AIP, after adjusting for potential confounders (OR: 0.46; 95% CI: 0.21, 0.97). The third quartile of hPDI was also associated with reduced odds of high-risk AIP compared to the lowest one, after adjusting for potential confounders (OR: 0.40; 95% CI: 0.18, 0.89). However, neither crude nor adjusted models did find any significant association between uPDI and high-risk AIP. The non-linear associations of all types of plant-based diet indices with MetS and AIP are shown in Figure 2. A significant non-linear association was observed between hPDI and MetS (*P*_non − linearity_ = 0.04).

**Table 2:** Multivariate adjusted odds ratio (OR) and 95% confidence interval (CI) for the association of energy-adjusted plant-based diets with metabolic syndrome and high risk AIP.

	**Quartile 1**	**Quartile 2**	**Quartile 3**	**Quartile 4**	***P*-trend**
**PDI**					
**Metabolic syndrome**					
Crude model	1	1.02 (0.61, 1,71)	0.70 (0.39, 1.28)	0.90 (0.53, 1.53)	0.46
Model I^a^	1	1.08 (0.62, 1.88)	0.61 (0.32, 1.18)	0.70 (0.39, 1.26)	0.09
Model II^b^	1	1.45 (0.70, 3.04)	0.99 (0.4, 2.49)	1.10 (0.50, 2.42)	0.92
Model III^c^	1	1.35 (0.61, 2.97)	0.94 (0.35, 2.54)	0.96 (0.41, 2.49)	0.70
**AIP (high risk)**					
Crude model	1	1.00 (0.62, 1.61)	0.84 (0.50, 1.41)	0.68 (0.41, 1.10)	0.07
Model I^a^	1	1.04 (0.63, 1.71)	0.91 (0.52, 1.60)	0.68 (0.41, 1.15)	0.10
Model II^b^	1	1.14 (0.58, 2.24)	0.85 (0.36, 1.98)	0.52 (0.25, 1.07)	0.04
Model III^c^	1	1.09 (0.54, 2.19)	0.78 (0.33, 1.86)	0.46 (0.21, 0.97)	0.02
**hPDI**					
**Metabolic syndrome**					
Crude model	1	1.02 (0.58, 1.79)	1.32 (0.75, 2.34)	1.50 (0.87, 2.59)	0.08
Model I^a^	1	0.82 (0.45, 1.48)	0.82 (0.44, 1.51)	0.74 (0.40, 1.38)	0.38
Model II^b^	1	0.83 (0.37, 1.86)	0.82 (0.36, 1.87)	0.70 (0.30, 1.65)	0.44
Model III^c^	1	0.47 (0.19, 1.15)	0.64 (0.26, 1.58)	0.43 (0.16, 1.12)	0.18
**AIP (high risk)**					
Crude model	1	0.72 (0.44, 1.17)	0.89 (0.53, 1.48)	0.80 (0.49, 1.31)	0.61
Model I^a^	1	0.75 (0.45, 1.25)	0.83 (0.48, 1.42)	0.81 (0.47, 1.39)	0.57
Model II^b^	1	0.72 (0.34, 1.49)	0.48 (0.22, 1.03)	0.72 (0.32, 1.61)	0.31
Model III^c^	1	0.55 (0.25, 1.19)	0.40 (0.18, 0.89)	0.57 (0.24, 1.33)	0.17
**uPDI**					
**Metabolic syndrome**					
Crude model	1	1.09 (0.64, 1.86)	0.96 (0.57, 1.62)	0.74 (0.42, 1.32)	0.27
Model I^a^	1	1.17 (0.67, 2.04)	0.99 (0.57, 1.74)	0.88 (0.47, 1.64)	0.61
Model II^b^	1	1.13 (0.52, 2.47)	1.69 (0.78, 3.66)	1.14 (0.48, 2.71)	0.48
Model III^c^	1	1.25 (0.53, 2.95)	2.39 (1.01, 5.66)	1.44 (0.56, 3.71)	0.21
**AIP (high risk)**					
Crude model	1	1.16 (0.71, 1.89)	1.08 (0.67, 1.74)	1.07 (0.65, 1.77)	0.88
Model I^a^	1	1.15 (0.69, 1.92)	0.89 (0.54, 1.47)	0.80 (0.47, 1.38)	0.30
Model II^b^	1	1.65 (0.80, 3.40)	0.94 (0.46, 1.91)	0.98 (0.45, 2.15)	0.64
Model III^c^	1	1.62 (0.77, 3.44)	0.99 (0.48, 2.08)	1.02 (0.45, 2.29)	0.74

a Adjusted for age, sex, and energy intake.

b Adjusted for age, sex, energy intake, education status, smoking status, marital status, SES, physical activity, margarine, and hydrogenated oil.

c Adjusted for age, sex, energy intake, education status, smoking status, marital status, SES, physical activity, margarine, hydrogenated oil, and BMI.

In a sensitivity analysis, we evaluated the association of plant-based diets with MetS, after excluding subjects who reported fruit and vegetable intake >1,000 g per day (Supplementary Table 3). The results showed that higher adherence to hPDI was related to a 73% decreased odds of MetS in the fully-adjusted model (OR_Q4 vs. Q1_: 0.27; 95% CI: 0.09, 0.77), and moderate adherence to uPDI was linked to an increased odds of MetS (OR_Q3 vs. Q1_: 2.79; 95% CI: 1.14, 6.81).

### Association of plant-based diets with components of MetS

The association of plant-based diets with components of MetS is presented in Table 3. Considering the first quartile of PDI as the reference group, higher adherence to PDI was not associated with any components of Mets in both crude and adjusted models. Subjects in the highest quartile of hPDI had lower odds of elevated fasting glucose compared to the first one in the fully-adjusted model (OR: 0.36; 95% CI: 0.13, 0.99). In addition, in Model I, those in the highest quartile of hPDI had 43% marginally significant decreased odds of hypertriglyceridemia (OR: 0.57; 95% CI: 0.32, 1.00). Participants in the third and fourth quartiles of uPDI had higher odds for hypertension after adjusting for potential confounders (OR_Q3 vs. Q1_: 3.38; 95% CI: 1.51, 7.57; OR_Q4 vs. Q1_: 2.42; 95% CI: 1.02, 5.75).

**Table 3:** Multivariate adjusted odds ratio (OR) and 95% confidence interval (CI) for the association of energy-adjusted plant-based diets with components of metabolic syndrome.

	**Quartile 1**	**Quartile 2**	**Quartile 3**	**Quartile 4**	***P*-trend**
**PDI**					
**Abdominal obesity**					
Model I^a^	1	1.09 (0.62, 1.90)	1.03 (0.55, 1.94)	0.94 (0.52, 1.68)	0.74
Model II^b^	1	1.17 (0.37, 3.74)	2.74 (0.60, 12.52)	2.75 (0.77, 9.87)	0.07
**High blood pressure**					
Model I^a^	1	1.23 (0.73, 2.08)	0.73 (0.40, 1.32)	0.91 (0.53, 1.57)	0.37
Model II^b^	1	0.90 (0.44, 1.83)	0.45 (0.18, 1.12)	1.04 (0.49, 2.22)	0.90
**High fasting glucose**					
Model I^a^	1	1.26 (0.66, 2.40)	0.82 (0.39, 1.72)	0.69 (0.35, 1.38)	0.13
Model II^b^	1	1.06 (0.46, 2.41)	1.00 (0.37, 2.76)	0.54 (0.21, 1.36)	0.16
**Hypertriglyceridemia**					
Model I^a^	1	0.84 (0.51, 1.40)	0.87 (0.49, 1.54)	0.82 (0.48, 1.39)	0.53
Model II^b^	1	0.82 (0.42, 1.60)	0.73 (0.32, 1.69)	0.66 (0.32, 1.35)	0.25
**Low HDL-C**					
Model I^a^	1	1.17 (0.54, 2.52)	0.80 (0.32, 2.03)	1.42 (0.65, 3.08)	0.49
Model II^b^	1	1.71 (0.62, 4.68)	0.99 (0.25, 3.96)	1.62 (0.55, 4.80)	0.58
**hPDI**					
**Abdominal obesity**					
Model I^a^	1	1.02 (0.58, 1.79)	0.92 (0.51, 1.68)	0.79 (0.43, 1.48)	0.42
Model II^b^	1	0.26 (0.06, 1.07)	0.35 (0.09, 1.37)	0.62 (0.15, 2.65)	0.65
**High blood pressure**					
Model I^a^	1	0.85 (0.50, 1.45)	0.93 (0.53, 1.65)	0.65 (0.36, 1.17)	0.20
Model II^b^	1	0.76 (0.35, 1.65)	0.96 (0.43, 2.18)	0.66 (0.28, 1.56)	0.49
**High fasting glucose**					
Model I^a^	1	0.75 (0.38, 1.48)	0.65 (0.32, 1.31)	0.57 (0.28, 1.17)	0.13
Model II^b^	1	0.38 (0.14, 0.98)	0.54 (0.21, 1.36)	0.36 (0.13, 0.99)	0.11
**Hypertriglyceridemia**					
Model I^a^	1	0.85 (0.51, 1.42)	0.87 (0.51, 1.51)	0.57 (0.32, 1.00)	0.07
Model II^b^	1	0.53 (0.25, 1.13)	0.52 (0.24, 1.12)	0.46 (0.20, 1.05)	0.08
**Low HDL-C**					
Model I^a^	1	0.82 (0.36, 1.90)	1.23 (0.53, 2.86)	1.52 (0.68, 3.44)	0.17
Model II^b^	1	0.62 (0.20, 1.96)	1.10 (0.35, 3.45)	1.23 (0.40, 3.84)	0.46
**uPDI**					
**Abdominal obesity**					
Model I^a^	1	1.41 (0.77, 2.58)	0.96 (0.55, 1.70)	0.82 (0.45, 1.50)	0.31
Model II^b^	1	0.99 (0.29, 3.49)	1.74 (0.48, 6.32)	0.40 (0.10, 1.69)	0.46
**High blood pressure**					
Model I^a^	1	0.99 (0.58, 1.69)	1.69 (0.99, 2.89)	1.22 (0.69, 2.17)	0.19
Model II^b^	1	0.85 (0.39, 1.85)	3.38 (1.51, 7.57)	2.42 (1.02, 5.75)	0.004
**High fasting glucose**					
Model I^a^	1	0.55 (0.29, 1.06)	0.74 (0.40, 1.38)	0.77 (0.39, 1.54)	0.58
Model II^b^	1	0.51 (0.21, 1.25)	0.75 (0.31, 1.77)	0.80 (0.31, 2.07)	0.75
**Hypertriglyceridemia**					
Model I^a^	1	1.22 (0.72, 2.08)	1.29 (0.77, 2.16)	1.07 (0.61, 1.88)	0.75
Model II^b^	1	1.66 (0.80, 3.45)	1.43 (0.70, 2.93)	1.33 (0.60, 2.95)	0.56
**Low HDL-C**					
Model I^a^	1	1.70 (0.80, 3.63)	1.19 (0.54, 2.64)	0.92 (0.38, 2.23)	0.71
Model II^b^	1	2.13 (0.73, 6.21)	1.98 (0.67, 5.81)	1.40 (0.41, 4.75)	0.55

a Adjusted for age, sex, and energy intake.

b Adjusted for age, sex, energy intake, education status, smoking status, marital status, SES, physical activity, margarine, hydrogenated oil, and BMI.

### Association of plant-based diets with serum level of adropin

Higher adherence to plant-based diet indices (per 1 quartile increment in PDI, hPDI, and uPDI) was not associated with serum level of adropin (as a continuous variable), after adjustment for potential confounders (B: 2.06, 95% CI: −0.88, 5.01; *P* = 0.17 for PDI; B: 0.57, 95% CI: −2.56, 3.70; *P* = 0.72 for hPDI; B: −0.46, 95% CI: −3.56, 2.63; *P* = 0.77 for uPDI) (Table 4).

**Table 4:** Linear association of energy-adjusted plant-based diet indices with serum level of adropin (as a continues variable).

	**B**	**95% CI**	***P***	***R* ^2^**
**Per 1 quartile of PDI**				
Crude model	0.71	−2.48, 3.89	0.66	-
Model I^a^	0.39	−2.91, 3.68	0.82	0.01
Model II^b^	2.09	−0.85, 5.03	0.16	0.02
Model III^c^	2.06	−0.88, 5.01	0.17	0.03
**Per 1 quartile of hPDI**				
Crude model	−1.14	−4.31, 2.03	0.48	0.001
Model I^a^	−0.73	−4.21, 2.76	0.68	0.01
Model II^b^	0.60	−2.53, 3.73	0.71	0.02
Model III^c^	0.57	−2.56, 3.70	0.72	0.02
**Per 1 quartile of uPDI**				
Crude model	−2.11	−5.39, 1.16	0.21	0.003
Model I^a^	−1.60	−5.07, 1.86	0.36	0.01
Model II^b^	−0.52	−3.62, 2.57	0.74	0.02
Model III^c^	−0.46	−3.56, 2.63	0.77	0.02

a Adjusted for age, sex, and energy intake.

b Adjusted for age, sex, energy intake, smoking status, and physical activity.

c Adjusted for age, sex, energy intake, smoking status, physical activity, and BMI.

## Discussion

This population-based cross-sectional study revealed that PDI and hPDI were not associated with MetS, whereas higher adherence to PDI and moderate adherence to hPDI decreased the odds of high-risk AIP in middle-aged adults. In addition, moderate adherence to uPDI was associated with greater odds of MetS, while no significant association was found between uPDI and high-risk AIP. Among MetS components, higher adherence to hPDI was detected to be associated with lower odds of hyperglycemia and hypertriglyceridemia, while higher adherence to uPDI was found to be associated with greater odds of hypertension. No significant association was found between plant-based diet indices and serum levels of adropin.

Previous studies suggested that plant-based diets, especially healthy plant foods, might have a role in the prevention and management of MetS and high-risk AIP, and unhealthy plant foods might increase the risk of diseases (23, 42–44). Our results suggested that hPDI might be associated with decreased odds of some components of MetS, and high-risk AIP and uPDI might be associated with an increased likelihood of overall MetS.

The current study found that higher adherence to PDI was not associated with overall MetS or its components. This finding is consistent with previous population-based studies that showed greater adherence to PDI was not associated with MetS and its components in the Iranian and South Korean populations (45, 46). In addition, a study conducted on a representative sample of Canadian adults demonstrated no significant association between plant-based diet indices and the incidence of CVD and mortality (47). Our findings suggested that greater adherence to hPDI was not associated with overall MetS, while it was associated with decreased odds of having hyperglycemia and hypertriglyceridemia. In agreement with our findings, several studies suggested that hPDI was not associated with overall MetS, abdominal obesity, hypertension, and low HDL-c (45, 46). However, the majority of previous studies, especially studies on western societies, suggested that the PDI and hPDI might decline the risk of MetS and its components (28, 30, 44, 48). To interpret the lack of association between PDI and hPDI with MetS and its components, several points should be taken into account. Compared to Western countries, the consumption of animal foods including red and processed meats is less common, and the consumption of plant foods including grains, potatoes, legumes, fruits, and vegetables is more common in the Asian population (49, 50). In other words, a significant percentage of energy intake comes from carbohydrates and starchy vegetables in the Asian population. These sources of energy could limit the ability of PDI and hPDI to change the metabolic response significantly and might therefore result in a null association between PDI and hPDI with MetS. In addition, the intake of fish in the highest quartile of plant-based diets was less than the lowest one. Fish intake could decrease the risk of MetS; therefore, it is possible that the low consumption of fish in Asian nations could interact with the effects of plant foods, especially fruits and vegetables (51, 52).

The majority of previous studies demonstrated that higher adherence to uPDI was associated with a greater risk of MetS and its components, especially in Western countries (27, 42, 43). However, several other studies, especially in the Asian population, did not observe any significant association between uPDI with MetS and its components (44, 45). In the current study, moderate adherence to uPDI (third quartile) was associated with elevated odds of MetS and in the highest quartile, and this association was not significant. In addition, moderate adherence to uPDI was associated with increased odds of hypertension, and in the highest quartile of uPDI, this association was attenuated. In the fourth quartile of uPDI, the intake of fruits, vegetables, fiber, nutrients, and antioxidants was low, while the intake of energy, carbohydrates, red and processed meats, and sodium was high. However, we expected that greater adherence to uPDI would be associated with higher odds of MetS (53–55). In this case, some points should be taken into account; high fruits and other plant-based foods, especially energy-rich plant foods, might elevate the prevalence of MetS, while in order to take advantage of the beneficial effect of fruits and vegetables on MetS, moderate consumption is suggested (56–58). In addition, heavy metals and chemical pesticide content of plant foods, especially vegetables, could be related to the prevalence of MetS (59, 60). In other words, the presence of pesticides and heavy metals in soil and plant foods was considered a concern in Iran (61–64). These issues might attenuate or change the association between different types of plant-based diet indices and MetS in our population.

A limited number of studies evaluated the association of dietary patterns, such as plant-based diet indices with AIP. Higher adherence to PDI and moderate adherence to hPDI were associated with lower odds of higher levels of AIP in the current study which was consistent with previous studies (22, 23). However, no significant association was observed between uPDI and AIP. These results might be related to the association between plant-based diet indices with triglycerides and HDL-c. Although PDI was not separately associated with triglycerides and HDL-c, it was associated with the logarithm of their ratio (AIP). Higher adherence to hPDI decreased the triglyceride levels; this reduction in triglycerides might have a role in decreased AIP. For uPDI, no significant association was observed for triglycerides, HDL-c, as well as AIP. A previous trial showed that the combination of the Mediterranean Diet (MD) and physical activity had a beneficial role in decreasing AIP levels (23). Two other studies reported that a snack rich in fiber and adherence to a healthy diet guideline were not significantly associated with AIP levels (22, 65). In addition, a meta-analysis demonstrated that total and saturated fat had no significant beneficial effect on serum triglyceride or HDL-c levels, as components of AIP (66). Another study suggested that there was a significant positive association between the quality of dietary fat and AIP (67). Furthermore, previous studies reported that low carbohydrate diets could decrease serum triglycerides and increase HDL-c levels (68, 69). However, the association of different types of plant-based diet indices with triglycerides and HDL-c, as the components of AIP, could predict the AIP levels. In addition, the independent effect of macronutrients on triglycerides and HDL-c, especially intake of carbohydrates and fats, should be considered in the interpretation of AIP levels.

To the best of our knowledge, there is no previous study that investigated the association of dietary patterns with adropin. The current study was the first investigation that evaluated the association between plant-based diet indices and adropin levels, although no significant association was found. Previous studies suggested that the intake of energy and macronutrients, including carbohydrates, proteins, and fats, might affect serum adropin levels (13, 70, 71). In addition, an experimental study demonstrated that a low-carbohydrate high-fat diet was associated with greater adropin levels, and a high-carbohydrate low-fat diet was associated with lower adropin levels in mice (21). Another investigation assessed the effect of dietary intake of sugars, including glucose, fructose, and high-fructose corn syrup (HFCS) on serum adropin levels. The results suggested that the intake of glucose decreased and the intake of fructose increased the adropin levels. However, HFCS intake did not change the adropin levels. In addition, the mentioned study reported that the effect of glucose and fructose intake on adropin was similar to their effect on serum triglycerides (72). However, the interaction between macronutrients and sugar intake in different types of plant-based diet indices, as well as the effect of confounders on serum levels of adropin, might be contributed to the association between plant-based diet indices and adropin levels.

### Strengths and limitations

This study was conducted on a large somehow representative sample of adults. However, the finding could be extrapolated to the general adult population. As a novelty, this study is the first one that evaluated the association of plant-based diet indices with AIP and serum adropin levels. The use of validated questionnaires and adjustments for potential confounders could be considered as the other strengths of the current study. However, some limitations should be considered. First, to evaluate the dietary intake, we applied a self-reported semi-quantitative FFQ that might enhance the measurement errors and misclassification of individuals. In addition, recall bias was another limitation of such a questionnaire that should be considered. Second, unknown and residual confounders should also be taken into account. Finally, because of the nature of cross-sectional studies, the causality of the association between exposures and outcomes could not clearly be determined.

## Conclusion

The current population-based cross-sectional study demonstrated that PDI and hPDI were not associated with MetS, while higher adherence to hPDI was associated with decreased odds of hyperglycemia and hypertriglyceridemia. In addition, moderate adherence to uPDI was associated with an increased prevalence of MetS and hypertension. In addition, high and moderate adherence to PDI and hPDI were associated with decreased odds of high-risk AIP, respectively. No significant association was found between plant-based diet indices and serum adropin levels. To confirm the findings of the current study and clearly determine causality, future studies with prospective design are warranted.

## Data availability statement

The raw data supporting the conclusions of this article will be made available by the authors, without undue reservation.

## Ethics statement

The studies involving human participants were reviewed and approved by the Ethics Committee of the Isfahan University of Medical Sciences (No: IR.MUI.RESEARCH.REC.1400.370). The patients/participants provided their written informed consent to participate in this study.

## Author contributions

FS, PS, KL, AF, GA, and SMS contributed to conception, design, data collection, data interpretation, manuscript drafting, and agreed on all aspects of the study. All authors contributed to the article and approved the submitted version.

## References

[1] Eckel RH Grundy SM Zimmet PZ. The metabolic syndrome. Lancet. (2005) 365:1415–28. 10.1016/S0140-6736(05)66378-7 15836891

[2] Dommermuth R Ewing K. Metabolic syndrome: Systems thinking in heart disease. Primary Care. (2018) 45:109–29. 10.1016/j.pop.2017.10.003 29406938

[3] Alberti G Zimmet P Shaw J Grundy SM. The IDF consensus worldwide definition of the metabolic syndrome. Brussels: International Diabetes Federation. (2006) 23:469–80. 10.1111/j.1464-5491.2006.01858.x 16681555

[4] Beltrán-Sánchez H Harhay MO Harhay MM McElligott S. Prevalence and trends of metabolic syndrome in the adult US population, 1999–2010. J Am Coll Cardiol. (2013) 62:697–703. 10.1016/j.jacc.2013.05.064 23810877 PMC3756561

[5] Vishram JK Borglykke A Andreasen AH Jeppesen J Ibsen H Jørgensen T. Impact of age and gender on the prevalence and prognostic importance of the metabolic syndrome and its components in Europeans. The MORGAM prospective cohort project. PLoS ONE. (2014) 9:e107294. 10.1371/journal.pone.0107294 25244618 PMC4171109

[6] Prasad D Kabir Z Dash A Das B. Prevalence and risk factors for metabolic syndrome in Asian Indians: A community study from urban Eastern India. J Cardiovasc Dis Res. (2012) 3:204–11. 10.4103/0975-3583.98895 22923938 PMC3425027

[7] Stepanova M Rafiq N Younossi ZM. Components of metabolic syndrome are independent predictors of mortality in patients with chronic liver disease: A population-based study. Gut. (2010) 59:1410–5. 10.1136/gut.2010.213553 20660697

[8] Wilson PW D'Agostino RB Parise H Sullivan L Meigs JB. Metabolic syndrome as a precursor of cardiovascular disease and type 2 diabetes mellitus. Circulation. (2005) 112:3066–72. 10.1161/CIRCULATIONAHA.105.539528 16275870

[9] Genser L Mariolo JRC Castagneto-Gissey L Panagiotopoulos S Rubino F. Obesity, type 2 diabetes, and the metabolic syndrome: Pathophysiologic relationships and guidelines for surgical intervention. Surg Clin. (2016) 96:681–701. 10.1016/j.suc.2016.03.013 27473795

[10] Fahed G Aoun L Bou Zerdan M Allam S Bou Zerdan M Bouferraa Y. Metabolic syndrome: Updates on pathophysiology and management in 2021. Int J Mol Sci. (2022) 23:786. 10.3390/ijms23020786 35054972 PMC8775991

[11] Ghadge AA Khaire AA. Leptin as a predictive marker for metabolic syndrome. Cytokine. (2019) 121:154735. 10.1016/j.cyto.2019.154735 31154250

[12] Kumari R Kumar S Kant R. An update on metabolic syndrome: Metabolic risk markers and adipokines in the development of metabolic syndrome. Diabet Metabol Syndr. (2019) 13:2409–17. 10.1016/j.dsx.2019.06.005 31405652

[13] Kumar KG Trevaskis JL Lam DD Sutton GM Koza RA Chouljenko VN. Identification of adropin as a secreted factor linking dietary macronutrient intake with energy homeostasis and lipid metabolism. Cell Metab. (2008) 8:468–81. 10.1016/j.cmet.2008.10.011 19041763 PMC2746325

[14] Yosaee S Khodadost M Esteghamati A Speakman JR Shidfar F Nazari MN. Metabolic syndrome patients have lower levels of adropin when compared with healthy overweight/obese and lean subjects. Am J Men's Health. (2017) 11:426–34. 10.1177/1557988316664074 27550773 PMC5675274

[15] Zang H Jiang F Cheng X Xu H Hu X. Serum adropin levels are decreased in Chinese type 2 diabetic patients and negatively correlated with body mass index. Endocr J. (2018) 65:685–91. 10.1507/endocrj.EJ18-0060 29669965

[16] Fan Z Zhang Y Zou F Xu T Pan P Hu C. Serum adropin level is associated with endothelial dysfunction in patients with obstructive sleep apnea and hypopnea syndrome. Sleep Breath. (2021) 25:117–23. 10.1007/s11325-020-02072-7 32253609

[17] Zhang X Li X Feng J Chen X. Association of metabolic syndrome with atherogenic index of plasma in an urban Chinese population: A 15-year prospective study. Nutr Metabol Cardiovasc Dis. (2019) 29:1214–9. 10.1016/j.numecd.2019.07.006 31378627

[18] Zhu X Yu L Zhou H Ma Q Zhou X Lei T. Atherogenic index of plasma is a novel and better biomarker associated with obesity: A population-based cross-sectional study in China. Lipids Health Dis. (2018) 17:1–6. 10.1186/s12944-018-0686-8 29506577 PMC5836428

[19] Niroumand S Khajedaluee M Khadem-Rezaiyan M Abrishami M Juya M Khodaee G. Atherogenic Index of Plasma (AIP): A marker of cardiovascular disease. Med J Islam Repub Iran. (2015) 29:240. 26793631 PMC4715400

[20] Onat A Can G Kaya H Hergenç G. “Atherogenic index of plasma” (log10 triglyceride/high-density lipoprotein–cholesterol) predicts high blood pressure, diabetes, and vascular events. J Clin Lipidol. (2010) 4:89–98. 10.1016/j.jacl.2010.02.005 21122635

[21] Ganesh Kumar K Zhang J Gao S Rossi J McGuinness OP Halem HH. Adropin deficiency is associated with increased adiposity and insulin resistance. Obesity. (2012) 20:1394–402. 10.1038/oby.2012.31 22318315 PMC3905465

[22] Edwards MK Loprinzi PD. Physical activity and diet on atherogenic index of plasma among adults in the United States: Mediation considerations by central adiposity. Eur J Clin Nutr. (2018) 72:826–31. 10.1038/s41430-017-0066-x 29321685

[23] Di Renzo L Cinelli G Dri M Gualtieri P Attinà A Leggeri C. Mediterranean personalized diet combined with physical activity therapy for the prevention of cardiovascular diseases in Italian women. Nutrients. (2020) 12:113456. 10.3390/nu12113456 33187188 PMC7697155

[24] Kastorini C-M Milionis HJ Esposito K Giugliano D Goudevenos JA Panagiotakos DB. The effect of Mediterranean diet on metabolic syndrome and its components: A meta-analysis of 50 studies and 534,906 individuals. J Am Coll Cardiol. (2011) 57:1299–313. 10.1016/j.jacc.2010.09.073 21392646

[25] Ghorabi S Salari-Moghaddam A Daneshzad E Sadeghi O Azadbakht L Djafarian K. Association between the DASH diet and metabolic syndrome components in Iranian adults. Diabet Metabol Syndr. (2019) 13:1699–704. 10.1016/j.dsx.2019.03.039 31235081

[26] Bell LK Edwards S Grieger JA. The relationship between dietary patterns and metabolic health in a representative sample of adult Australians. Nutrients. (2015) 7:6491–505. 10.3390/nu7085295 26251918 PMC4555134

[27] Kim H Lee K Rebholz CM Kim J. Plant-based diets and incident metabolic syndrome: Results from a South Korean prospective cohort study. PLoS Med. (2020) 17:e1003371. 10.1371/journal.pmed.1003371 33206633 PMC7673569

[28] McGrath L Fernandez M-L. Plant-based diets and metabolic syndrome: Evaluating the influence of diet quality. J Agri Food Res. (2022) 9:100322. 10.1016/j.jafr.2022.100322

[29] Satija A Bhupathiraju SN Rimm EB Spiegelman D Chiuve SE Borgi L. Plant-based dietary patterns and incidence of type 2 diabetes in US men and women: Results from three prospective cohort studies. PLoS Med. (2016) 13:e1002039. 10.1371/journal.pmed.1002039 27299701 PMC4907448

[30] Satija A Bhupathiraju SN Spiegelman D Chiuve SE Manson JE Willett W. Healthful and unhealthful plant-based diets and the risk of coronary heart disease in US adults. J Am Coll Cardiol. (2017) 70:411–22. 10.1016/j.jacc.2017.05.047 28728684 PMC5555375

[31] Ko B-J Park KH Shin S Zaichenko L Davis CR Crowell JA. Diet quality and diet patterns in relation to circulating cardiometabolic biomarkers. Clin Nutr. (2016) 35:484–90. 10.1016/j.clnu.2015.03.022 25912185 PMC4596724

[32] Park KH Zaichenko L Peter P Davis CR Crowell JA Mantzoros CS. Diet quality is associated with circulating C-reactive protein but not irisin levels in humans. Metabolism. (2014) 63:233–41. 10.1016/j.metabol.2013.10.011 24315778 PMC4373656

[33] Mirmiran P Esfahani FH Mehrabi Y Hedayati M Azizi F. Reliability and relative validity of an FFQ for nutrients in the Tehran lipid and glucose study. Public Health Nutr. (2010) 13:654–62. 10.1017/S1368980009991698 19807937

[34] Willett WC Howe GR Kushi LH. Adjustment for total energy intake in epidemiologic studies. Am J Clin Nutr. (1997) 65:1220S−8S. 10.1093/ajcn/65.4.1220S 9094926

[35] Hu FB Stampfer MJ Rimm E Ascherio A Rosner BA Spiegelman D. Dietary fat and coronary heart disease: A comparison of approaches for adjusting for total energy intake and modeling repeated dietary measurements. Am J Epidemiol. (1999) 149:531–40. 10.1093/oxfordjournals.aje.a009849 10084242

[36] Flack JM Adekola B. Blood pressure and the new ACC/AHA hypertension guidelines. Trends Cardiovasc Med. (2020) 30:160–4. 10.1016/j.tcm.2019.05.003 31521481

[37] Dobiásová M Frohlich J. The plasma parameter log (TG/HDL-C) as an atherogenic index: Correlation with lipoprotein particle size and esterification rate inapob-lipoprotein-depleted plasma (FERHDL). Clin Biochem. (2001) 34:583–8. 10.1016/S0009-9120(01)00263-6 11738396

[38] Alberti KG Eckel RH Grundy SM Zimmet PZ Cleeman JI Donato KA. Harmonizing the metabolic syndrome: A joint interim statement of the international diabetes federation task force on epidemiology and prevention; national heart, lung, and blood institute; American heart association; world heart federation; international atherosclerosis society; and international association for the study of obesity. Circulation. (2009) 120:1640–5. 10.1161/CIRCULATIONAHA.109.192644 19805654

[39] Craig C Marshall A Sjostrom M Bauman A Lee P Macfarlane D. International physical activity questionnaire-short form. J Am Coll Health. (2017) 65:492–501.28641040

[40] Mokhtari E Mirzaei S Asadi A Akhlaghi M Saneei P. Association between plant-based diets and metabolic health status in adolescents with overweight and obesity. Sci Rep. (2022) 12:1–12. 10.1038/s41598-022-17969-4 35962035 PMC9374681

[41] Hu FB Rimm E Smith-Warner SA Feskanich D Stampfer MJ Ascherio A. Reproducibility and validity of dietary patterns assessed with a food-frequency questionnaire. Am J Clin Nutr. (1999) 69:243–9. 10.1093/ajcn/69.2.243 9989687

[42] Satija A Malik V Rimm EB Sacks F Willett W Hu FB. Changes in intake of plant-based diets and weight change: Results from 3 prospective cohort studies. Am J Clin Nutr. (2019) 110:574–82. 10.1093/ajcn/nqz049 31127828 PMC6735841

[43] Kim H Rebholz CM Garcia-Larsen V Steffen LM Coresh J Caulfield LE. Operational differences in plant-based diet indices affect the ability to detect associations with incident hypertension in middle-aged US adults. J Nutr. (2020) 150:842–50. 10.1093/jn/nxz275 31722418 PMC7138677

[44] Gómez-Donoso C Martínez-González MÁ Martínez JA Gea A Sanz-Serrano J Perez-Cueto FJA. A provegetarian food pattern emphasizing preference for healthy plant-derived foods reduces the risk of overweight/obesity in the SUN Cohort. Nutrients. (2019) 11:1553. 10.3390/nu11071553 31324022 PMC6683267

[45] Amini MR Shahinfar H Djafari F Sheikhhossein F Naghshi S Djafarian K. The association between plant-based diet indices and metabolic syndrome in Iranian older adults. Nutr Health. (2021) 27:435–44. 10.1177/0260106021992672 33626298

[46] Kim H Lee K Rebholz CM Kim J. Association between unhealthy plant-based diets and the metabolic syndrome in adult men and women: A population-based study in South Korea. Br J Nutr. (2021) 125:577–90. 10.1017/S0007114520002895 32713361

[47] Lazarova SV Sutherland JM Jessri M. Adherence to emerging plant-based dietary patterns and its association with cardiovascular disease risk in a nationally representative sample of Canadian adults. Am J Clin Nutr. (2022) 2022:2104. 10.23889/ijpds.v7i3.2104 35265975 PMC9257478

[48] Qian F Liu G Hu FB Bhupathiraju SN Sun Q. Association between plant-based dietary patterns and risk of type 2 diabetes: A systematic review and meta-analysis. J Am Med Assoc Intern Med. (2019) 179:1335–44. 10.1001/jamainternmed.2019.2195 31329220 PMC6646993

[49] Micha R Khatibzadeh S Shi P Andrews KG Engell RE Mozaffarian D. Global, regional and national consumption of major food groups in 1990 and 2010: A systematic analysis including 266 country-specific nutrition surveys worldwide. Br Med J Open. (2015) 5:e008705. 10.1136/bmjopen-2015-008705 26408285 PMC4593162

[50] Daniel CR Cross AJ Koebnick C Sinha R. Trends in meat consumption in the USA. Public Health Nutr. (2011) 14:575–83. 10.1017/S1368980010002077 21070685 PMC3045642

[51] Baik I Abbott RD Curb JD Shin C. Intake of fish and n-3 fatty acids and future risk of metabolic syndrome. J Am Diet Assoc. (2010) 110:1018–26. 10.1016/j.jada.2010.04.013 20630158

[52] Zaribaf F Falahi E Barak F Heidari M Keshteli AH Yazdannik A. Fish consumption is inversely associated with the metabolic syndrome. Eur J Clin Nutr. (2014) 68:474–80. 10.1038/ejcn.2014.5 24549028

[53] Wei B Liu Y Lin X Fang Y Cui J Wan J. Dietary fiber intake and risk of metabolic syndrome: A meta-analysis of observational studies. Clin Nutr. (2018) 37:1935–42. 10.1016/j.clnu.2017.10.019 29137803

[54] Parks EJ Hellerstein MK. Carbohydrate-induced hypertriacylglycerolemia: Historical perspective and review of biological mechanisms. Am J Clin Nutr. (2000) 71:412–33. 10.1093/ajcn/71.2.412 10648253

[55] Strazzullo P D'Elia L Kandala N-B Cappuccio FP. Salt intake, stroke, and cardiovascular disease: Meta-analysis of prospective studies. Br Med J. (2009) 339:bmj.b4567. 10.1136/bmj.b4567 19934192 PMC2782060

[56] Stanhope KL Havel PJ. Fructose consumption: Potential mechanisms for its effects to increase visceral adiposity and induce dyslipidemia and insulin resistance. Curr Opin Lipidol. (2008) 19:16. 10.1097/MOL.0b013e3282f2b24a 18196982 PMC4151171

[57] Park S Ham J-O Lee B-K. Effects of total vitamin A, vitamin C, and fruit intake on risk for metabolic syndrome in Korean women and men. Nutrition. (2015) 31:111–8. 10.1016/j.nut.2014.05.011 25466654

[58] Xia Y Gu Y Yu F Zhang Q Liu L Meng G. Association between dietary patterns and metabolic syndrome in Chinese adults: A propensity score-matched case-control study. Sci Rep. (2016) 6:1–8. 10.1038/srep34748 27708414 PMC5052517

[59] Haverinen E Fernandez MF Mustieles V Tolonen H. Metabolic syndrome and endocrine disrupting chemicals: An overview of exposure and health effects. Int J Environ Res Public Health. (2021) 18:13047. 10.3390/ijerph182413047 34948652 PMC8701112

[60] Planchart A Green A Hoyo C Mattingly CJ. Heavy metal exposure and metabolic syndrome: Evidence from human and model system studies. Curr Environ Health Rep. (2018) 5:110–24. 10.1007/s40572-018-0182-3 29460222 PMC6053628

[61] Faraji M Alizadeh I Oliveri Conti G Mohammadi A. Investigation of health and ecological risk attributed to the soil heavy metals in Iran: Systematic review and meta-analysis. Sci Tot Environ. (2023) 857:158925. 10.1016/j.scitotenv.2022.158925 36174699

[62] Fakhri Y Bjørklund G Bandpei AM Chirumbolo S Keramati H Hosseini Pouya R. Concentrations of arsenic and lead in rice (*Oryza sativa* L.) in Iran: A systematic review and carcinogenic risk assessment. Food Chem Toxicol. (2018) 113:267–77. 10.1016/j.fct.2018.01.018 29341878

[63] Moosazadeh M Khanjani N. Human contamination with organochlorine pesticides in Iran: A systematic review. Health Dev J. (2015) 4.35439599

[64] Tavakoly Sany SB. The occurrence and toxicity of dioxins and dioxin-like polychlorinated biphenyls in foodstuffs collected from different cities of Iran: A systematic review. J Nutr Fast Health. (2022) 10:51–9.

[65] Sunarti S Rini SLS Sinorita H Ariani D. Effect of fiber-rich snacks on C-reactive protein and atherogenic index in type 2 diabetes patients. Roman J Diabet Nutr Metabol Dis. (2018) 25:271–6. 10.2478/rjdnmd-2018-0042

[66] Yu-Poth S Zhao G Etherton T Naglak M Jonnalagadda S Kris-Etherton PM. Effects of the National Cholesterol Education Program's Step I and Step II dietary intervention programs on cardiovascular disease risk factors: A meta-analysis. Am J Clin Nutr. (1999) 69:632–46. 10.1093/ajcn/69.4.632 10197564

[67] Moussavi Javardi MS Madani Z Movahedi A Karandish M Abbasi B. The correlation between dietary fat quality indices and lipid profile with Atherogenic index of plasma in obese and non-obese volunteers: A cross-sectional descriptive-analytic case-control study. Lipids Health Dis. (2020) 19:1–9. 10.1186/s12944-020-01387-4 32979928 PMC7519513

[68] Dong T Guo M Zhang P Sun G Chen B. The effects of low-carbohydrate diets on cardiovascular risk factors: A meta-analysis. PLoS ONE. (2020) 15:e0225348. 10.1371/journal.pone.0225348 31935216 PMC6959586

[69] Ha K Kim K Chun OK Joung H Song Y. Differential association of dietary carbohydrate intake with metabolic syndrome in the US and Korean adults: Data from the 2007–2012 NHANES and KNHANES. Eur J Clin Nutr. (2018) 72:848–60. 10.1038/s41430-017-0031-8 29339830

[70] Stevens JR Kearney ML St-Onge MP Stanhope KL Havel PJ Kanaley JA. Inverse association between carbohydrate consumption and plasma adropin concentrations in humans. Obesity. (2016) 24:1731–40. 10.1002/oby.21557 27460714 PMC5184848

[71] St-Onge MP Shechter A Shlisky J Tam CS Gao S Ravussin E. Fasting plasma adropin concentrations correlate with fat consumption in human females. Obesity. (2014) 22:1056–63. 10.1002/oby.20631 24115373 PMC3968187

[72] Butler AA St-Onge M-P Siebert EA Medici V Stanhope KL Havel PJ. Differential responses of plasma adropin concentrations to dietary glucose or fructose consumption in humans. Sci Rep. (2015) 5:14691. 10.1038/srep14691 26435060 PMC4592955

