# Supplementary material for: Association of plant-based diets with adropin, atherogenic index of plasma, and metabolic syndrome and its components: A cross-sectional study on adults
Source: Front Nutr. 2023 Apr 11;10:1077709. doi: 10.3389/fnut.2023.1077709 (PMC10128915; doi:10.3389/fnut.2023.1077709)
Supplement: Supplementary file 1 [file Table_1.DOCX]

| **Supplemental Table 1**. Scoring criteria for the plant-based diets indices. | | | | |
| --- | --- | --- | --- | --- |
| Plant food groups | Items | PDI | hPDI | uPDI |
| **Healthy** | | | | |
| Whole grains | Iranian dark breads, barley | Positive | Positive | Reverse |
| Fruits | Melon, honeydew melon, watermelon, pear, apricot, cherry, apple, peach, nectarine, Japanese plum, ﬁg, grape, kiwifruit, grapefruit, orange, persimmon, tangerine, pomegranate, date, plum, sour cherry, strawberry, banana, lime, sweet lemon, pineapple, cranberry, mulberry, Dried ﬁg, raisin, dried mulberry, dehydrated peach, dehydrated apricot | Positive | Positive | Reverse |
| Vegetables | Lettuce, tomato, cucumber, fresh basil, mixed vegetables, squash, eggplant, celery, green peas, green beans, carrot, garlic, onion, cabbage, spinach, bell pepper, mushroom, turnip | Positive | Positive | Reverse |
| Nuts | Peanuts, almonds, walnuts, pistachios, hazelnuts, sunﬂower seeds | Positive | Positive | Reverse |
| Legumes | Lentils, kidney beans, chickpeas, broad beans, soy beans, mung beans, split peas | Positive | Positive | Reverse |
| Vegetable oils | Vegetable oils, olive oil, green olives | Positive | Positive | Reverse |
| Tea and coffee | Tea, coffee | Positive | Positive | Reverse |
| **Less healthy** | | | | |
| Fruit juices | Grapefruit juice, orange juice, apple juice, melon juice, lemon juice | Positive | Reverse | Positive |
| Refined grains | White breads, French bread, white rice, spaghetti, vermicelli, noodles, wheat ﬂour, biscuits, crackers | Positive | Reverse | Positive |
| Potatoes | Boiled potatoes, potato chips, French fries | Positive | Reverse | Positive |
| Sugar sweetened beverages | Soft drinks | Positive | Reverse | Positive |
| Sweets and desserts | Mufﬁns, other cakes, sugar, white granulated sugar, honey, jam, gaz (Iranian sweet), hard candy, chocolates, caramel ﬂan, donuts, canned fruits, | Positive | Reverse | Positive |
| **Animal foods** | | | | |
| Animal fat | Butter, animal fats | Reverse | Reverse | Reverse |
| Dairy | Skim milk, low-fat milk, low-fat yogurt, kashk, high-fat milk, whole chocolate and cocoa milk, high-fat yogurt, creamy yogurt, cream cheese, other cheese, cream, chocolate ice cream, vanilla ice cream, yogurt drink | Reverse | Reverse | Reverse |
| Eggs | Eggs | Reverse | Reverse | Reverse |
| Fish | Canned tuna ﬁsh, other ﬁsh | Reverse | Reverse | Reverse |
| Meat | Lamb, veal, ground lamb, beef sausages, hamburger, poultry without skin, poultry with skin, organs meat | Reverse | Reverse | Reverse |
| Miscellaneous animal-based foods | Pizza, mayonnaise | Reverse | Reverse | Reverse |

| **Supplemental Table 2.** Multivariate adjusted intake of nutrients across energy-adjusted quartiles of the plant-based dietary indices. | | | | | | | | | | |
| --- | --- | --- | --- | --- | --- | --- | --- | --- | --- | --- |
| **Variables** |  | **PDI** | |  | **hPDI** | |  | **uPDI** | |  |
|  |  | **Quartile 1** | **Quartile 4** | **P-value ^a^** | **Quartile 1** | **Quartile 4** | **P-value ^a^** | **Quartile 1** | **Quartile 4** | **P-value ^a^** |
| **Intake of nutrients** | | | | | | | | | | |
| Energy (kcal)^b^ | | 2592.73±60.62 | 2191.44±54.91 | <0.001 | 2130.57±63.42 | 2528.70±56.81 | <0.001 | 2303.07±60.77 | 2542.59±61.67 | <0.001 |
| Carbohydrate (gr) | | 311.45±4.21 | 370.38±3.72 | <0.001 | 325.92±4.57 | 361.06±4.15 | <0.001 | 326.40±4.32 | 364.79±4.46 | <0.001 |
| Protein (gr) | | 93.14±1.42 | 71.34±1.26 | <0.001 | 84.64±1.60 | 77.34±1.45 | 0.01 | 90.54±1.45 | 73.88±1.49 | <0.001 |
| Fat (gr) | | 76.81±1.63 | 62.52±1.44 | <0.001 | 74.24±1.69 | 64.77±1.54 | <0.001 | 73.71±1.59 | 61.58±1.65 | <0.001 |
| Dietary fiber (gr) | | 17.53±0.58 | 23.42±0.51 | <0.001 | 17.28±0.56 | 25.47±0.51 | <0.001 | 24.41±0.55 | 17.79±0.57 | <0.001 |
| Cholesterol (mg) | | 357.34±9.90 | 207.03±8.76 | <0.001 | 317.16±10.96 | 238.96±9.95 | <0.001 | 350.10±9.88 | 212.73±10.20 | <0.001 |
| SAF (gr) | | 27.41±0.67 | 18.27±0.60 | <0.001 | 25.60±0.72 | 19.89±0.66 | <0.001 | 24.90±0.70 | 20.03±0.72 | <0.001 |
| MUFA (gr) | | 25.17±0.61 | 19.30±0.54 | <0.001 | 23.99±0.64 | 19.90±0.58 | <0.001 | 23.70±0.61 | 19.86±0.63 | <0.001 |
| PUFA (gr) | | 15.42±0.69 | 16.76±0.61 | 0.46 | 15.66±0.70 | 16.77±0.64 | 0.61 | 16.00±0.66 | 14.02±0.68 | 0.01 |
| Oleic acid (gr) | | 22.70±0.68 | 18.99±0.61 | 0.01 | 21.69±0.70 | 19.89±0.64 | 0.16 | 22.31±0.66 | 18.53±0.69 | 0.01 |
| Linoleic fat (gr) | | 12.76±0.67 | 13.79±0.60 | 0.67 | 13.00±0.68 | 13.54±0.62 | 0.93 | 12.54±0.65 | 11.99±0.67 | 0.03 |
| Linolenic fat (gr) | | 0.35±0.02 | 0.32±0.02 | 0.63 | 0.37±0.02 | 0.24±0.02 | <0.001 | 0.34±0.02 | 0.34±0.02 | 0.64 |
| EPA (gr) | | 0.02±0.002 | 0.01±0.001 | <0.001 | 0.02±0.002 | 0.01±0.001 | 0.26 | 0.02±0.002 | 0.01±0.002 | <0.001 |
| DHA (gr) | | 0.06±0.004 | 0.03±0.004 | <0.001 | 0.05±0.005 | 0.04±0.004 | 0.22 | 0.07±0.004 | 0.02±0.004 | <0.001 |
| Manganese (mg) | | 3.21±0.16 | 4.35±0.15 | <0.001 | 3.32±0.17 | 4.08±0.15 | 0.01 | 4.36±0.16 | 3.52±0.17 | 0.01 |
| Zinc (mg) | | 11.67±0.25 | 8.97±0.22 | <0.001 | 10.62±0.27 | 9.82±0.24 | 0.19 | 11.89±0.24 | 8.90±0.24 | <0.001 |
| Copper (mg) | | 1.38±0.03 | 1.56±0.03 | <0.001 | 1.41±0.03 | 1.55±0.03 | 0.01 | 1.64±0.03 | 1.34±0.03 | <0.001 |
| Iron (mg) | | 17.43±0.38 | 18.07±0.34 | 0.63 | 17.64±0.39 | 17.75±0.35 | 0.85 | 16.73±0.36 | 19.75±0.37 | <0.001 |
| Magnesium (mg) | | 274.83±6.19 | 290.27±5.48 | 0.15 | 261.32±6.17 | 304.44±5.60 | <0.001 | 329.5.39 | 240.21±5.57 | <0.001 |
| Calcium (mg) | | 1079.69±34.37 | 820.89±30.42 | <0.001 | 869.75±35.83 | 974.02±32.53 | 0.20 | 1088.59±32.77 | 760.26±33.84 | <0.001 |
| Vitamin E (mg) | | 6.50±0.30 | 7.01±.26 | 0.56 | 7.04±0.30 | 6.68±0.27 | 0.80 | 7.43±0.29 | 6.39±0.29 | 0.07 |
| Folate (mcg) | | 311.08±10.40 | 362.71±9.29 | 0.01 | 299.34±10.54 | 373.53±9.57 | <0.001 | 408.06±9.48 | 274.19±9.79 | <0.001 |

^a^ Resulted from ANCOVA for quantitative variables (mean±SE).
^b^ Energy intake was adjusted for age and sex; other nutrient intake adjusted for age, sex, and energy intake.

| **Supplemental Table 3.**Multivariate adjusted odds ratio (OR) and 95% confidence interval (CI) for the association of energy-adjusted plant-based diets with metabolic syndrome, after excluding subjects who were reported fruits and vegetable intake greater than 1000 gr/day). | | | | | | | | | | |
| --- | --- | --- | --- | --- | --- | --- | --- | --- | --- | --- |
|  |  | **PDI** | | | | | | | |  |
|  |  | **Quartile 1** |  | **Quartile 2** |  | **Quartile 3** |  | **Quartile 4** |  | **P-trend** |
| Crude model |  | 1 |  | 1.33 (0.76, 2.35) |  | 0.96 (0.54, 1.70) |  | 0.91 (0.51, 1.65) |  | 0.54 |
| Model I ^1^ |  | 1 |  | 1.28 (0.70, 2.34) |  | 0.83 (0.45, 1.53) |  | 0.75 (0.40, 1.40) |  | 0.20 |
| Model II ^2^ |  | 1 |  | 1.34 (0.61, 2.93) |  | 1.02 (0.44, 2.38) |  | 0.84 (0.35, 2.00) |  | 0.60 |
| Model III ^3^ |  | 1 |  | 1.32 (0.57, 3.08) |  | 1.07 (0.43, 2.65) |  | 0.75 (0.29, 1.92) |  | 0.49 |
|  |  | **hPDI** | | | | | | | |  |
| Crude model |  | 1 |  | 0.91 (0.49, 1.67) |  | 1.16 (0.66, 2.04) |  | 0.99 (0.55, 1.81) |  | 0.78 |
| Model I ^1^ |  | 1 |  | 0.75 (0.40, 1.43) |  | 0.75 (0.41, 1.40) |  | 0.50 (0.25, 0.98) |  | 0.06 |
| Model II ^2^ |  | 1 |  | 0.95 (0.40, 2.27) |  | 0.72 (0.31, 1.66) |  | 0.40 (0.16, 1.05) |  | 0.06 |
| Model III ^3^ |  | 1 |  | 0.58 (0.22, 1.54) |  | 0.52 (0.21, 1.30) |  | 0.27 (0.09, 0.77) |  | 0.02 |
|  |  | **uPDI** | | | | | | | |  |
| Crude model |  | 1 |  | 1.12 (0.63, 2.01) |  | 1.09 (0.63, 1.89) |  | 0.83 (0.46, 1.51) |  | 0.60 |
| Model I ^1^ |  | 1 |  | 1.18 (0.64, 2.17) |  | 1.18 (0.66, 2.12) |  | 0.91 (0.48, 1.76) |  | 0.90 |
| Model II ^2^ |  | 1 |  | 1.64 (0.70, 3.87) |  | 1.91 (0.85, 4.26) |  | 1.36 (0.55, 3.38) |  | 0.34 |
| Model III ^3^ |  | 1 |  | 2.03 (0.79, 5.20) |  | 2.79 (1.14, 6.81) |  | 1.82 (0.67, 4.91) |  | 0.12 |
| ^1^ Adjusted for age, sex, and energy intake. ^2^ Adjusted for age, sex, energy intake, education status, smoking status, marital status, SES, physical activity, margarine, and hydrogenated oil. ^3^ Adjusted for age, sex, energy intake, education status, smoking status, marital status, SES, physical activity, margarine, hydrogenated oil, and BMI. | | | | | | | | | | |
